# The Sortase A Substrates FnbpA, FnbpB, ClfA and ClfB Antagonize Colony Spreading of *Staphylococcus aureus*

**DOI:** 10.1371/journal.pone.0044646

**Published:** 2012-09-07

**Authors:** Eleni Tsompanidou, Emma L. Denham, Mark J. J. B. Sibbald, Xiao-mei Yang, Jolien Seinen, Alexander W. Friedrich, Girbe Buist, Jan Maarten van Dijl

**Affiliations:** 1 Department of Medical Microbiology, University of Groningen and University Medical Center Groningen, Groningen, The Netherlands; 2 Department of Pharmaceutical Biology, University of Groningen, Groningen, The Netherlands; 3 Key Laboratory of Molecular Virology, Shanghai Medical College, Fudan University, Shanghai, China; University of Iowa Carver College of Medicine, United States of America

## Abstract

*Staphylococcus aureus* is an important human pathogen that is renowned both for its rapid transmission within hospitals and the community, and for the formation of antibiotic resistant biofilms on medical implants. Recently, it was shown that *S. aureus* is able to spread over wet surfaces. This motility phenomenon is promoted by the surfactant properties of secreted phenol-soluble modulins (PSMs), which are also known to inhibit biofilm formation. The aim of the present studies was to determine whether any cell surface-associated *S. aureus* proteins have an impact on colony spreading. To this end, we analyzed the spreading capabilities of strains lacking non-essential components of the protein export and sorting machinery. Interestingly, our analyses reveal that the absence of sortase A (SrtA) causes a hyper-spreading phenotype. SrtA is responsible for covalent anchoring of various proteins to the staphylococcal cell wall. Accordingly, we show that the hyper-spreading phenotype of *srtA* mutant cells is an indirect effect that relates to the sortase substrates FnbpA, FnbpB, ClfA and ClfB. These surface-exposed staphylococcal proteins are known to promote biofilm formation, and cell-cell interactions. The hyper-spreading phenotype of *srtA* mutant staphylococcal cells was subsequently validated in *Staphylococcus epidermidis*. We conclude that cell wall-associated factors that promote a sessile lifestyle of *S. aureus* and *S. epidermidis* antagonize the colony spreading motility of these bacteria.

## Introduction

*Staphylococcus aureus* is an opportunistic human pathogen that is currently a leading cause of infections throughout the world. This Gram-positive bacterium can cause a wide variety of both acute and chronic diseases ranging from superficial skin infections to life-threatening endocarditis and sepsis [1], [2]. The ability of *S. aureus* to cause these infections is due to the production of secreted and cell wall-associated virulence factors that are coordinately expressed. These factors include proteins that are necessary for host colonization, invasion, biofilm formation, toxicogenesis, immune evasion or spreading throughout the host.

To sort proteins to their correct extracytoplasmic locations, Gram-positive bacteria have several pathways for protein targeting and transport. *S. aureus* contains at least six of these pathways [3]. Most proteins, including virulence factors are translocated across the cytoplasmic membrane via the Sec pathway. These proteins are synthesized in the cytoplasm with an N-terminal Sec-type signal peptide that directs them to the Sec translocase, which is embedded in the membrane [3]–[7]. The Sec translocase can only facilitate the membrane passage of proteins in an unfolded state [8], [9]. Upon translocation, type I signal peptidases cleave the signal peptide to liberate the proteins from the membrane. Various folding catalysts can then assist the folding of the translocated proteins into their active and protease-resistant conformation [3], [10]–[12]. Some proteins that are translocated via the Sec pathway are retained in the membrane or cell wall. When a translocated protein lacks a specific signal for retention in these subcellular compartments, it is usually secreted into the extracellular milieu [3], [13]. Proteins can be bound to the cell wall either in a non-covalent manner via specific binding domains, or covalently through the enzymatic activity of so-called sortases.

Gram-positive bacteria employ sortases to covalently link exported proteins with a special C-terminal LPxTG motif to the cell wall. These sortases are membrane-bound transpeptidases that cleave the peptide bond between the Thr and Gly residues of the LPxTG motif, and catalyze the formation of an amide bond between the carboxyl group of the Thr residue and the free amino end of a pentaglycine cross bridge in peptidoglycan precursors [14]–[19]. The sortase A (SrtA) enzyme from *S. aureus* is a prototypical member of the sortase family [20]–[23]. *S. aureus* strains lacking the *srtA* gene are unable to retain and display LPxTG proteins at the cell surface. As a consequence, *srtA* mutant strains are defective in the establishment of acute infections [21].

There are 19 staphylococcal proteins that carry a C-terminal LPxTG motif and 2 that carry a C-terminal LPxAG motif [3], [13], [24]–[26]. These include protein A (Spa), two fibronectin-binding proteins (FnbpA and FnbpB) [27], two clumping factors (ClfA and ClfB), three cell wall-anchored proteins with large serine-aspartate repeat domains (SdrC, SdrD and SdrE) [28], a collagen-binding protein (Can), a plasmin-sensitive protein (Pls) [29], FmtB [30], and eleven staphylococcal surface (Sas) proteins. For some of these proteins a direct role in biofilm formation has been reported. This applies to Spa [31], [32], FnbpA and FnbpB [33]–[36].

We have previously shown that *S. aureus* cells can employ secreted phenol–soluble modulins (PSMs) for their translocation over wet surfaces. At the same time, certain PSMs are very effective in preventing biofilm formation [37]. The PSMs thus seem to have a decisive role in the transitions between sessile and motile lifestyles of *S. aureus*. While the role of secreted PSMs in spreading motility has been established, it was so far not known whether any cell-associated factors are also involved in this process. Therefore, the primary aim of the present studies was to identify cell-associated factors that impact on spreading motility. As a first approach to find out whether any cell surface-associated proteins may be involved in spreading, we investigated spreading motility of mutant strains lacking non-essential components of the protein export and sorting machinery. Interestingly, this revealed that *srtA* mutant cells are more efficient spreaders than the corresponding parental strains. Further analyses showed that this relates to the spreading-limiting roles of the sortase substrates FnbpA, FnbpB, ClfA and ClfB.

## Results and Discussion

**Figure 1:**
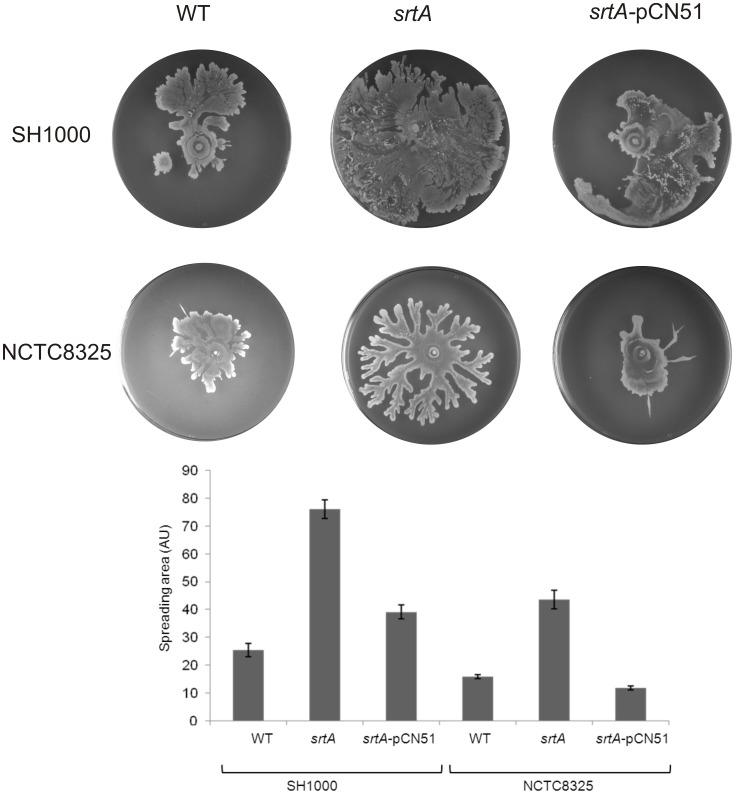
Hyper-spreading phenotype of *srtA* mutant *S. aureus* strains. From an overnight culture, an aliquot of 2 µl was spotted in the middle of a TSA plate, which was then incubated overnight at 37°C. The analyses include the laboratory strains *S. aureus* SH1000 and NCTC8325 (both labeled WT), as well as their *srtA* mutant derivatives (labeled *srtA*) and *srtA* mutants complemented with a plasmid pCN51-borne copy of *S. aureus srtA* (labeled *srtA*-pCN51). The spreading areas of the investigated mutant and parental strains were determined by ImageJ. The graphs show the areas covered in arbitrary units (AU) and respective standard deviations.

The requirement for non-essential protein secretion machinery components in colony spreading by *S. aureus* was assessed by testing the secretion mutants listed in Table S1 for their ability to spread on TSA soft agar plates. Of all tested strains, only the *srtA* mutant showed a significant change in spreading. Intriguingly, this strain displayed an enhanced colony spreading phenotype as is shown in Figure 1. This spreading phenotype of the *srtA* mutant was completely reversed to the wild-type phenotype by ectopic expression of *srtA* from the plasmid *srtA*-pCN51 (Fig. 1).

**Figure 2:**
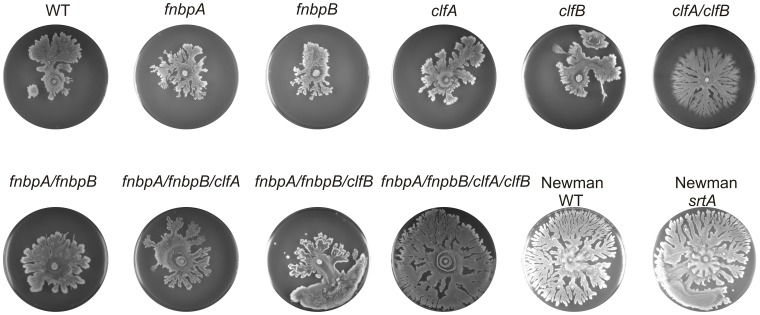
The influence of *fnbpA*, *fnbpB*, *clfA* and *clfB* mutations on colony spreading of *S. aureus.* Spreading motility of *S. aureus* SH1000-derived *fnbpA*, *fnbpB*, *clfA* and/or *clfB* mutant strains or the *S. aureus* Newman *srtA* mutant strain was assayed as described for Figure 1.

The *srtA* mutant strains are unable to link LPxTG proteins covalently to the cell wall and, because of this, they are attenuated in virulence. This suggested that the effect of the *srtA* mutation on spreading would also be an indirect consequence of the absence of cell wall coupling of one or more LPxTG proteins. Many LPxTG proteins belong to the MSCRAMMs (microbial surface components recognizing adhesive matrix molecules) and promote bacterial attachment to the extracellular matrix of host tissues. Some of these MSCRAMMs, such as FnbpA and FnbpB, have been implicated in biofilm formation and other MSCRAMMs, such as ClfA and ClfB, have been implicated in cell-cell interactions. Since spreading motility on the one hand and biofilm formation or cell aggregation on the other hand are processes with opposite effects, we investigated whether the individual deletion of the *fnbpA*, *fnbpB*, *clfA* or *clfB* genes would result in enhanced spreading. None of these single mutant strains had a major impact on colony spreading, although the *fnbpA*, *fnbpB* and *clfB* mutant cells did cover slightly, but statistically significantly larger areas than the corresponding parental strain or *clfA* mutant cells (Fig. 2; Table S2). Since this suggested that the absence of only one of these proteins might not be sufficient for an increased spreading phenotype, double, triple and quadruple mutant strains were constructed that lacked *fnbpA*, *fnpbB*, *clfA* and/or *clfB*. As shown in Figure 2, the mutant lacking all four of these genes showed the most strongly enhanced spreading phenotype that was comparable to the phenotype of the *srtA* single mutant strain (for statistical evaluation, see Table S2). As shown with the double or triple mutant strains, the four individual mutations had additive effects in enhancing colony spreading. Thus, the two fibronectin-binding proteins FnbpA and FnbpB and the two clumping factors ClfA and ClfB counteract spreading. While we cannot exclude the possibility that other LPxTG proteins also counteract spreading, the observed effect of the quadruple *fnbpA fnbpB clfA clfB* mutation is fully sufficient to explain the hyper-spreading phenotype of the *srtA* mutant. It should be noted that FnbpA, FnbpB, ClfA and ClfB do not block colony spreading as evidenced by the spreading of the parental strains used in the present studies as well as a range of clinical isolates that readily spread on soft agar [38]. Thus, it seems that in the absence of FnbpA, FnbpB, ClfA and ClfB the cells are less tightly associated with each other and, consequently, they can cover larger areas on soft agar plates by means of their spreading motility.

The FnbpA, FnbpB, ClfA and ClfB proteins can promote adhesion of *S. aureus* cells to a variety of molecules and surfaces and they have been implicated in cell-cell adhesion. In relation to our present findings, it is interesting to note that the *fnpbB* gene is less common in highly virulent *S. aureus* isolates, and that the presence of *fnbpB* is associated with reduced transmission of staphylococcal skin infections in a rabbit model [39], [40]. This seems to suggest that spreading activity and transmission of *S. aureus* could perhaps be linked. Furthermore, FnbpA is a highly variable surface protein. The *fnbpA* gene has a mosaic structure, which indicates that this gene is evolving not only through point mutations, but also through recombination events [41]. ClfA mediates attachment to plasma clots, to platelets and to plastic biomaterial used for medical implants. Lastly, ClfB promotes cell clumping in the presence of fibrinogen. However, ClfB is not only able to bind to fibrinogen itself, but also to proteins present in the envelope of squamous cells and to desquamated nasal epithelial cells [26], [42], [43]. Interestingly, the production of FnbpA, FnbpB, ClfA and ClfB in different *S. aureus* strains seems to be highly variable [38], [44], [45]. This may at least partly explain our previous observation that the spreading abilities of different *S. aureus* clinical isolates are highly variable [38], [44]. This view is further supported by the observation that strain Newman, which produces truncated forms of FnbpA and FnbpB, is a very efficient spreader (Fig. 2). These truncated FnbPs are no longer anchored to the cell surface but secreted, which leads to a loss of their function [46]. In fact the high spreading activity of strain Newman is comparable to that of the *srtA* mutant or the *fnbpA fnbpB clfA clfB* quadruple mutant derivative of strain SH1000 (Fig. 2). Notably, the mutations in *fnbpA* and *fnbpB* may not be sufficient to explain the increased spreading of strain Newman, but our previous studies suggest that this strain also produces very low levels of ClfB, if any [44]. This may contribute to the hyper-spreading phenotype of strain Newman. Consistent with these considerations, a *srtA* deletion increased the spreading capacity of strain Newman only slightly (Figure 2; Table S2). This could be due to impaired cell wall-binding of ClfA and perhaps also low-levels of ClfB. However, we cannot completely exclude the possibility that impaired cell wall-binding of other LPxTG proteins, such as Protein A, might add to the hyper-spreading phenotype of the *srtA* mutant of strain Newman.

Depending on the strain and growth condition, the *fnbpA* and *fnbpB* genes are negatively regulated by the Agr system [47]–[49]. On the other hand, the Agr system positively regulates the synthesis of PSMs that are critical for spreading motility [38]. The differential Agr-regulated production of FnbpAB and the PSMs is thus fully compatible with our present findings that FnbpAB counteract spreading. Though the *clfA* and *clfB* genes are not regulated by Agr, but by SarA, they are highly expressed during the early exponential growth phase and barely during the late exponential or stationary growth phases [47], [50], [51]. The production of ClfA and ClfB thus correlates positively with that of FnbpAB and negatively with PSM production, which is also fully consistent with the presently observed negative role of ClfA and ClfB in spreading.

**Figure 3:**
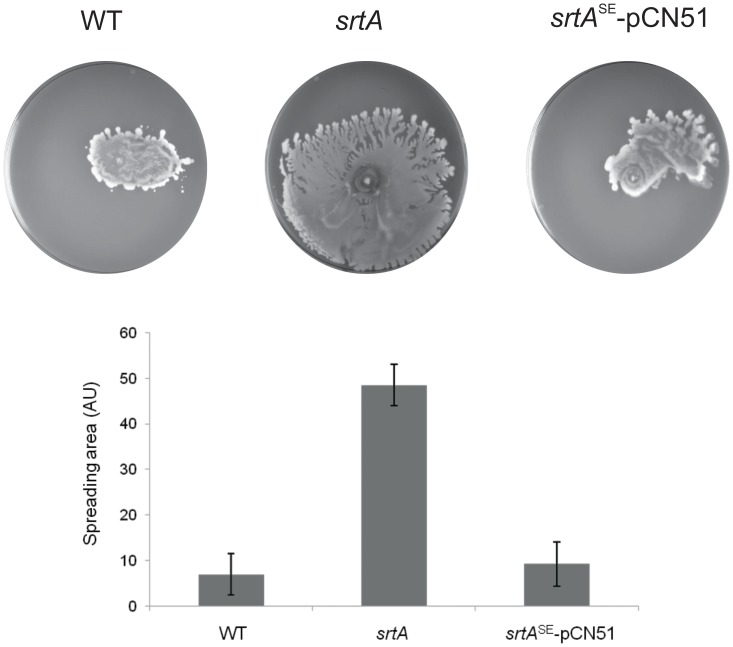
Hyper-spreading phenotype of a *srtA* mutant of *S. epidermidis* 1457. Spreading motility of *S. epidermidis* 1457 (WT), a *srtA* mutant derivative of this strain (*srtA*), and a complemented derivative of the *srtA* mutant (*srtA^Se^*-pCN51) was assayed as described for Figure 1.

Lastly, to investigate whether surface-attached proteins also set a limit to spreading motility in other staphylococci, we turned to *Staphylococcus epidermidis*. This bacterium is renowned for its high capacity to form biofilms on medical implants [52]. Nevertheless, *S. epidermidis* does produce phenol-soluble modulins [53], [54], which should provide it with an intrinsic capacity for spreading motility. As shown in Figure 3, wild-type cells of *S. epidermidis* strain 1457 did indeed spread on soft agar plates, albeit to a lesser extent than cells of *S. aureus* SH1000. As predicted on the basis of our experiments with *S. aureus*, the *srtA* mutant of *S. epidermidis* displayed a massively increased spreading over soft agar plates (Fig. 3). Furthermore, this hyper-spreading phenotype of the *S. epidermidis srtA* mutant was completely reversed to the low-level spreading of the parental strain upon ectopic expression of the *S. epidermidis srtA* gene from plasmid *srtA^Se^*-pCN51 (Fig. 3). We therefore conclude that, also in *S. epidermidis*, the sortase-mediated cell wall anchoring of proteins sets a limit to spreading motility. Thus, this seems to be a conserved feature of staphylococcal spreading motility, which is fully consistent with the previously shown role of covalently anchored cell wall proteins in the formation of biofilms.

## Materials and Methods

### Bacterial Strains and Growth Conditions

The bacterial strains and plasmids that were used in the present studies are listed in Table S1. All *Escherichia coli* strains were grown in Luria-Bertani broth (LB) at 37°C under shaking conditions. *S. aureus* and *S. epidermidis* strains were grown in tryptic soy broth (TSB) at 37°C under vigorous shaking. Where necessary, ampicillin 100 µg/ml (for *E. coli*) or erythromycin 5 µg/ml (for *S. aureus* and *S. epidermidis*) were added to the growth medium.

### Construction of *S. aureus* and *S. epidermidis* Mutant Strains

The *S. aureus* and *S. epidermidis* mutants lacking secretion machinery genes (Table S1) were constructed using the temperature-sensitive plasmid pMAD [55] and previously described procedures [56]. All primers used are listed in Table S3. To delete particular genes, primer pairs with the designations F1/R1 and F2/R2 were used for PCR amplification of the respective upstream and downstream regions (each ∼500 bp). Primers R1 and F2 contain an overlap of 24 nucleotides, which served to fuse the amplified ‘front’ and ‘back’ flanking regions by PCR. The fused flanking regions were cloned in pMAD, and the resulting plasmids were used to delete the genes between these flanking regions from the *S. aureus* or *S. epidermidis* genome. To this end, the pMAD plasmids carrying the flanking regions were used to transform *S. aureus* strain RN4220 via electroporation. Next, these plasmids were isolated from the RN4220 strain and used to transform electrocompetent cells of *S. aureus* SH1000, NCTC8325 and Newman, or *S. epidermidis* 1457. Upon chromosomal plasmid insertion and excision, white colonies on plates with 80 µg/ml 5-bromo-4-chloro-3-indolyl-β-D-galactopyranoside were screened for the correct gene deletion by colony PCR using primers F1 and R2. To delete the *clfA* or *clfB* genes from the *S. aureus* SH1000 genome, the respective allelic replacements with antibiotic resistance markers were transferred from the original strains provided by T.J. Foster to the SH1000 strain by transduction with phage φ85 [57], [58].

### Complementation of the *srtA* Mutation

For complementation studies, the *srtA* genes of *S. aureus* and *S. epidermidis* were cloned in plasmid pCN51. Expression of a cloned gene in this plasmid is controlled by a cadmium-inducible promoter. The primers used for the amplification of the *srtA* genes are listed in Table S3 and the restriction sites used for cloning in pCN51 are shown in italics. The resulting plasmids *srtA*-pCN51 and *srtA^Se^*-pCN51 were used to transform electrocompetent *S. aureus* RN4220 cells, and the transformed cells were plated on TSA plates containing erythromycin. The restriction-modified plasmids were isolated from *S. aureus* RN4220 and then used to transform electrocompetent *S. aureus* SH1000 Δ*srtA*, *S. aureus* NCTC8325 Δ*srtA*, or *S. epidermidis* 1457 Δ*srtA*.

### Colony Spreading Assay

The colony spreading assay was performed as described by Kaito *et al* [59], with minor modifications. Briefly, TSB broth supplemented with 0.24% agar was used to prepare TSA soft agar plates. Each plate (10 ml) was dried for approximately 10 min in a laminar flow cabinet for optimal colony spreading conditions. From a TSB overnight culture of the strain to be tested for spreading, an aliquot of 2 µl was spotted in the centre of a TSA plate and the plates were then dried for an additional 5 min. Lastly, upon overnight incubation of the plates at 37°C, the spreading zones were examined and pictures were taken. To induce *srtA* expression from pCN51, soft agar plates were supplemented with 0.25 µM CdSO_4_. All spreading assays were repeated at least five times.

## Supporting Information

Table S1 **Bacterial strains and plasmids used in the present studies.**
(DOCX)

Table S2 **Statistical analysis of colony spreading by the different mutant strains.** The spreading areas of the investigated mutant and parental strains were determined by ImageJ. The Table show the areas covered in arbitrary units (AU). P-values were determined by the non-parametric Mann–Whitney *U* test.
(DOCX)

Table S3 **Primers used in the present studies.** Overlapping nucleotides are shown in bold; restriction sites in primers are underlined. ^a^These primers have an overlap with the kanamycin resistance cassette from pDG783.
(DOCX)
