# Supplementary material for: The Sortase A Substrates FnbpA, FnbpB, ClfA and ClfB Antagonize Colony Spreading of Staphylococcus aureus
Source: PLoS One. 2012 Sep 7;7(9):e44646. doi: 10.1371/journal.pone.0044646 (PMC3436756; doi:10.1371/journal.pone.0044646)
Supplement: Table S1 — Bacterial strains and plasmids used in the present studies. (DOCX) [file pone.0044646.s001.docx]

**Supplementary** **Table S1 Bacterial strains and plasmids used in the present studies**

| **Strains** | **Genotype** | **Reference** |
| --- | --- | --- |
| ***E. coli*** |  |  |
| *E. coli* DH5α | λ^-^ φ80d*lac*ΖΔΜ15 Δ(*lacZYA*-*argF*)U169 *recA1* *endA* *hsdR17*(^r^k^- m^k^-^)*sup*E44 *thi-1* *gyrA* *relA1* | [1] |
| *E. coli* TOP10 | Cloning host for TOPO vector; F^-^ *mc*rA Δ(*mrr*-*hsdRMS*-*mcrBC*) λ^-^ φ80d*lac*ΖΔΜ15 Δ*lacX74* *recA1* *araD*139 Δ(*ara*-*leu*)7697 *galU galK* *rps*L (Str^r^) *endA*1 *nupG* | Invitrogen Life Technologies |
| ***S. aureus*** |  |  |
| RN4220 | Restriction-deficient derivative of NCTC8325, cured of all known prophages | [2] |
| SH1000 | NCTC8325-4 derivative, *rsbU*^+^, *agr*^+^ | [3] |
| SH1000Δ*cidA::km* | *cidA*, Km^r^ | This work |
| SH1000 Δ*dsbA* | *dsbA* | This work |
| SH1000 Δ*lgt* | *lgt* | This work |
| SH1000 Δ*lrgA::km* | *lrgA*, Km^r^ | This work |
| SH1000 Δ*lspA* | *lspA* | This work |
| SH1000 Δ*prsA* | *prsA* | This work |
| SH1000 Δ*secG* | *secG* | [4] |
| SH1000 Δ*secY2* | *secY2* | [4] |
| SH1000 Δ*spsA* | *spsA* | This work |
| SH1000 Δ*srtA* | *srtA* | This work |
| SH1000 Δ*srtB* | *srtB* | This work |
| SH1000 Δ*tatA* | *tatA* | This work |
| SH1000 Δ*tatC::km* | *tatC*, Km^r^ | This work |
| SH1000 Δ*tatAC* | *tatA* *tatC* | This work |
| SH1000 Δ*mscL* | *mscL* | This work |
| SH1000 Δ*fnbpA* | *fnbpA* | This work |
| SH1000 Δ*fnbpB* | *fnbpB* | This work |
| SH1000 Δ*fnbpA* Δ*fnbpB* | *fnbpA, fnbpB* | This work |
| SH1000 Δ*clfA* | *clfA* | [5] |
| SH1000 Δ*clfB* | *clfB* | [5] |
| SH1000 Δ*clfA* Δ*clfB* | *clfA, clfB* | This work |
| SH1000 Δ*fnbpA* Δ*fnbpB,* Δ*clfA* | *fnbpA, fnbpB, clfA* | This work |
| SH1000 Δ*fnbpA* Δ*fnbpB,* Δ*clfB* | *fnbpA, fnbpB, clfB* | This work |
| SH1000 Δ*fnbpA* Δ*fnbpB* Δ*clfA* Δ*clfB* | *fnbpA, fnbpB, clfA, clfB* | [5] |
| NCTC8325 | HA-MSSA strain, *agr*^+^, *rsbU*^-^ | [6] |
| NCTC8325 Δ*srtA* | *srtA* | This work |
| Newman | NCTC 8178 clinical isolate | [7] |
| Newman Δ*srtA* | *srtA* | This work |
| ***S. epidermidis*** |  |  |
| 1457 | Biofilm positive strain | [8] |
| 1457 Δ*srtA* | *srtA* | This work |
| **Plasmids** | **Relevant properties** | **Reference** |
| pUC18 | Ap^r^, ColE1, φ80d*lac*Ζ; *lac* promoter | [9] |
| TOPO | pCR-Blint II-TOPO vector; Km^r^ | Invitrogen Life Technologies |
| pCN51 | *E. coli*/*S. aureus* shuttle vector that contains a cadmium-inducible promoter; Ap^r^ Em^r^ | [10] |
| *srtA*-pCN51 | pCN51 with *S. aureus srtA* gene; Ap^r^ Em^r^ | This work |
| *srtA^Se^*-pCN51 | pCN51 with *S. epidermidis srtA* gene; Ap^r^ Em^r^ | This work |

**References**

1. Hanahan D. (1983) Studies on transformation of *Escherichia coli* with plasmids. J Mol Biol 166: 557-580.

2. Kreiswirth BN, Lofdahl S, Betley MJ, O'Reilly M, Schlievert PM, et al. (1983) The toxic shock syndrome exotoxin structural gene is not detectably transmitted by a prophage. Nature 305: 709-712.

3. Horsburgh MJ, Aish JL, White IJ, Shaw L, Lithgow JK, et al. (2002) sigmaB modulates virulence determinant expression and stress resistance: Characterization of a functional rsbU strain derived from *Staphylococcus aureus* 8325-4. J Bacteriol 184: 5457-5467.

4. Sibbald MJ, Winter T, van der Kooi-Pol MM, Buist G, Tsompanidou E, et al. (2010) Synthetic effects of secG and secY2 mutations on exoproteome biogenesis in *Staphylococcus aureus*. J Bacteriol 192: 3788-3800.

5. Greene C, McDevitt D, Francois P, Vaudaux PE, Lew DP, et al. (1995) Adhesion properties of mutants of *Staphylococcus aureus* defective in fibronectin-binding proteins and studies on the expression of *fnb* genes. Mol Microbiol 17: 1143-1152.

6. Novick R. (1967) Properties of a cryptic high-frequency transducing phage in *Staphylococcus aureus*. Virology 33: 155-166.

7. Duthie ES, Lorenz LL. (1952) Staphylococcal coagulase; mode of action and antigenicity. J Gen Microbiol 6: 95-107.

8. Mack D, Siemssen N, Laufs R. (1992) Parallel induction by glucose of adherence and a polysaccharide antigen specific for plastic-adherent *Staphylococcus epidermidis*: Evidence for functional relation to intercellular adhesion. Infect Immun 60: 2048-2057.

9. Norrander J, Kempe T, Messing J. (1983) Construction of improved M13 vectors using oligodeoxynucleotide-directed mutagenesis. Gene 26: 101-106.

10. Charpentier E, Anton AI, Barry P, Alfonso B, Fang Y, et al. (2004) Novel cassette-based shuttle vector system for Gram-positive bacteria. Appl Environ Microbiol 70: 6076-6085.
