# Supplementary material for: The Sortase A Substrates FnbpA, FnbpB, ClfA and ClfB Antagonize Colony Spreading of Staphylococcus aureus
Source: PLoS One. 2012 Sep 7;7(9):e44646. doi: 10.1371/journal.pone.0044646 (PMC3436756; doi:10.1371/journal.pone.0044646)
Supplement: Table S2 — Statistical analysis of colony spreading by the different mutant strains. The spreading areas of the investigated mutant and parental strains were determined by ImageJ. The Table show the areas covered in arbitrary units (AU). P-values were determined by the non-parametric Mann–Whitney U test. (DOCX) [file pone.0044646.s002.docx]

**Supplementary Table S2. Statistical analysis of colony spreading by the different mutant strains**

| **Strain** | **Median** | **Minimum** | **Maximum** | **P-value** |
| --- | --- | --- | --- | --- |
| SH1000 WT | 23.7530 | 23.5843 | 23.9478 |  |
| SH1000 Δ*fnbpA* | 38.8829 | 30.3507 | 39.5127 | 0.05 |
| SH1000 Δ*fnbpB* | 33.1683 | 27.5171 | 39.4217 | 0.034 |
| SH1000 Δ*clfA* | 22.0563 | 20.3516 | 22.4481 | 0.05 |
| SH1000 Δ*clfB* | 26.7783 | 25.6002 | 29.8190 | 0.05 |
| SH1000 Δ*fnbpA*Δ*fnbpB* | 35.1047 | 31.5653 | 42.7399 | 0.05 |
| SH1000 Δ*clfA*Δ*clfB* | 31.2235 | 27.7190 | 33.3749 | 0.05 |
| SH1000 Δ*fnbpA*Δ*fnbpB*Δ*clfA* | 26.0380 | 23.9212 | 35.6289 | 0.127 |
| SH1000 Δ*fnbpA*Δ*fnbpB*Δ*clfB* | 39.9876 | 31.3127 | 45.1090 | 0.05 |
| SH1000 Δ*fnbpA*Δ*fnbpB*Δ*clfA*Δ*clfB* | 71.2227 | 61.0000 | 85.2500 | 0.05 |
| Newman WT | 51.1730 | 47.5035 | 53.5689 |  |
| Newman Δ*srtA* | 53.6421 | 50.4587 | 54.5466 | 0.275 |

The spreading areas of the investigated mutant and parental strains were determined by ImageJ. The Table show the areas covered in arbitrary units (AU). P-values were determined by the non-parametric Mann–Whitney *U* test.
