# Supplementary material for: The Sortase A Substrates FnbpA, FnbpB, ClfA and ClfB Antagonize Colony Spreading of Staphylococcus aureus
Source: PLoS One. 2012 Sep 7;7(9):e44646. doi: 10.1371/journal.pone.0044646 (PMC3436756; doi:10.1371/journal.pone.0044646)
Supplement: Table S3 — Primers used in the present studies. Overlapping nucleotides are shown in bold; restriction sites in primers are underlined. aThese primers have an overlap with the kanamycin resistance cassette from pDG783. (DOCX) [file pone.0044646.s003.docx]

**Supplementary Table S3. Primers used in the present studies**

| **Primer** | **Sequence (5’→3’)** |
| --- | --- |
| *cidA*-F1 | AATAAAGACTTTTACTTGAAT |
| *cidA*-R1*^a^* | **TTAGAACTCCAATTCACCCATGGCCCCC**GCGCCATCCCTTTCTAAATA |
| *cidA*-F2*^a^* | **CCGCAACTGTCCATACCATGGCCCCC**TGATTACGTGCAAGCCTTATTAAT |
| *cidA*-R2 | GATAGAAGATTCAAATCTTCC |
| *dsbA*-F1 | ATTTCTTTGGATATTTATATT |
| *dsbA*-R1 | **CTACGTCAGTCAGTCACCATGGCA**AATAACTCCTATTCATAT |
| *dsbA*-F2 | **TGCCATGGTGACTGACTGACG**TAGTCTTAATTGTTGAGATCA |
| *dsbA*-R2 | CTTTCGTTATAGTTTTCCCAC |
| *lgt*-F1 | GGTGTTGGTGTACTAATTACC |
| *lgt*-R1 | **CTACGTCAGTCAGTCACCATGGCA**TCAACCTACTCCTCACTCTTA |
| *lgt*-F2 | **TGCCATGGTGACTGACTGACG**TAGTGATAGTTTGAGGAAATTTTT |
| *lgt*-R2 | ACATTATTATTCTTTTGCGCC |
| *lrgA*-F1 | TAAAGCCAAAGATGATAATAA |
| *lrgA*-R1*^a^* | **TTAGAACTCCAATTCACCCATGGCCCCC**GCCTCCTACGTTTGATTTAA |
| *lrgA*-F2*^a^* | **CCGCAACTGTCCATACCATGGCCCCC**TAACCACTTAGCACTAAACACACC |
| *lrgA*-R2 | GTAATTCGGAAAAGCTTTAAG |
| *lspA*-F1 | CCAATTAAGTGTAGACGATTC |
| *lspA*-R1 | **TTACGTCAGTCAGTCACCATGGCA**TTTCGTTCCTCCAATCAATCG |
| *lspA*-F2 | **TGCCATGGTGACTGACTGACG**TAATGGAGACTTATGAATTTAACA |
| *lspA*-R2 | CGATATATTTTCTTTTAACAG |
| *prsA*-F1 | GAAAATGGCTTATATTCTATA |
| *prsA*-R1 | **TTACGTCAGTCAGTCACCATGGCA**AGTTGAAACTCCTTTGTAAGT |
| *prsA*-F2 | **TGCCATGGTGACTGACTGACG**TAACACAAAACCGAGCGACCGTGG |
| *prsA*-R2 | TTTGTTATATAGTGGTATTAT |
| *secA2-F1* | GTATAAAAGCATGCGGGTGAC |
| *secA2-R1^a^* | TTAGAACTCCAATTCACCCATGGCCCCCTTACTTCCCCACCATTCAGTT |
| *secA2-F2^a^* | CGCAACTGTCCATACCATGGCCCCCTAAATGAAAAGGGGTAGCGCATGA |
| *secA2-R2* | GTCGCATATATAATTTCGCTT |
| *secG-F1* | TTAAAACAGGACGCTTTATTG |
| *secG-R1* | TTACGTCAGTCAGTCACCATGGCAAAATTGTCCTCCGTTCCTTAT |
| *secG-F2* | TGCCATGGTGACTGACTGACGTAAGGTCCGGCGATGTAAATGTCG- |
| *secG-R2* | GCGTGCATATTCTAAAAAGCC |
| *secY2-F1* | TGTCTGGTTCACAAAGCATTT |
| *secY2-R1* | TTACGTCAGTCAGTCACCATGGCAGTTGCACCTCTTTTATATCAA |
| *secY2-F2* | TGCCATGGTGACTGACTGACGTAAGGAGGTAATTATGAAATACTT |
| *secY2-R2* | GCCTCTCCCTGATCATCAAAA |
| *spsA-F1* | TAGAGCTATAATTCCAGTATT |
| *spsA-R1* | TTACGTCAGTCAGTCACCATGGCAGATGTCACTCCTTTTTCGATC |
| *spsA-F2* | TGCCATGGTGACTGACTGACGTAAAAAGAGGTGTCAAAATTGAAA |
| *spsA-R2* | CCAACAATTTGGTCTTCATCA |
| *srtA-F1* | AATGGTGTAGTAATTGACTAG |
| *srtA-R1* | TTACGTCAGTCAGTCACCATGGCAACGTTAAGGCTCCTTTTATAC |
| *srtA-F2* | TGCCATGGTGACTGACTGACGTAATCTATTACGCTAATGGATGAA- |
| *srtA-R2* | CTCACATTACTTACTATTAAT |
| *srtB-F1* | TGAAAATATGGAGCGACGTAT |
| *srtB-R1* | TTACGTCAGTCAGTCACCATGGCAAAAAATCCTCTTTTATTAACG |
| *srtB-F2* | TGCCATGGTGACTGACTGACGTAAACAGAAAAGAGGATAATTATG |
| *srtB-R2* | ATCAAAATGATATAATTGATG |
| *tatA-F1* | TTATGGCATTTACATTATCTG |
| *tatA-R1* | CTACGTCAGTCAGTCACCATGGCAGATAATCAACCTCACTCATAA |
| *tatA-F2* | TGCCATGGTGACTGACTGACGTAGCACTGACCACACCTTACTGGT |
| *tatA-R2* | GACCCATAAATAATATTGGTA |
| *tatC-F1* | TGATGAAATGGCTGAAGCTGG |
| *tatC-R1^a^* | TTAGAACTCCAATTCACCCATGGCCCCCAAAATTTTTACTAACCGATG |
| *tatC-F2^a^* | CCGCAACTGTCCATACCATGGCCCCCTAACCTTATACGAATCAATGCTGT |
| *tatC-R2* | CGATTAGTAATGGTAATTTGG |
| *kan-F1* | GGGGGCCATGGGTGAATTGGAGTTCGTCTTG |
| *kan-R1* | GGGGGCCATGGTATGGACAGTTGCGGATGTA |
| *secA2-F3* | CGGAATTCGAATCCAGTACGATTTTTAG |
| *secA2-R3* | CGGGATCCTCCCGGTAACATACGACCTG |
| *srtA^Se^-F1* | AACTTTGTTCTTTAGCGTAACGAAT |
| *srtA^Se^-R1* | **TGCCATGGTGACTGACTGACGTAA**TTATGTTACTCCTTTATATTTATT |
| *srtA^Se^-F2* | **TTACGTCAGTCAGTCACCATGGCA**TATTCTTATAAGTGAAAGATACGTA |
| *srtA^Se^-R2* | CTTTATAGATGACTGCTCCAT |
| *srtA-CN 5’* | CAGCCGGATCCAATGTATAAAAGGAGCCTTAACGT |
| *srtA-CN 3’* | CGGAATTCTTATTTGACTTCTGTAGCTACAAA |
| *srtA^Se^-CN 5’* | CAGCCGGATCCAATGTATAAAAGGAGCCTTAACGTATGAAGCAGTGGATGAATAGA |
| *srtA^Se^-CN 3’* | CG GAATTCTTAGTTAATTTGTGTAGCTATGAA |

*Overlapping nucleotides are shown in bold; restriction sites in primers are underlined*

^a^ *These primers have an overlap with the kanamycin resistance cassette from pDG783*
